# Surveillance colonoscopy in PSC-IBD: Some answers but more questions remain

**DOI:** 10.1055/a-2514-9742

**Published:** 2025-02-06

**Authors:** Nilanga Nishad, Mo Thoufeeq, Sreedhar Subramanian

**Affiliations:** 1 7318 Gastroenterology, Sheffield Teaching Hospitals NHS Foundation Trust, Sheffield, United Kingdom of Great Britain and Northern Ireland; 2 2153 Cambridge University Hospitals NHS Foundation Trust, Cambridge, United Kingdom of Great Britain and Northern Ireland



We commend Rodrigo et al. on their recent study that demonstrates the superiority of dye-based chromoendoscopy (CE) compared with white light endoscopy (WLE) for neoplasia detection in patients with primary sclerosing cholangitis-inflammatory and bowel disease (PSC-IBD), reinforcing SCENIC guidelines 1 . It is now established that CE performs better in IBD surveillance 2 , but such evidence is sparse for PSC-IBD and, therefore, deserves investigation. However, we would like to highlight some aspects that deserve further discussion. Data on endoscopist volume and experience are not presented and previous studies have demonstrated this to be an important determinant of colonoscopy quality 3 4 . Information on the number of biopsies taken, withdrawal times, and presence of pseudo-polyps were also lacking. In addition, some patients may have had intermittent or persistent mucosal inflammation, an important driver of colorectal cancer (CRC) risk in IBD and comparable data in PSC, therefore, would be of considerable interest 5 . Inclusion of complete-cases-only analysis may have introduced some bias. Although the authors used multivariable logistic regression to control for confounders, differences in baseline characteristics, such as family history of CRC, might have skewed the findings, and propensity adjustment using inverse probability weighing might have circumvented this limitation.

The study's finding of higher neoplasia detection with CE underscores the value of technique over resolution, because high-definition WLE did not significantly outperform standard definition after adjustment. The potential for CE to detect subtle serrated lesions remains noteworthy. Despite compelling evidence of superiority for CE in IBD surveillance, it remains poorly adopted, but recent improvements such as foot-pump dye application may improve CE uptake. Future studies should focus on emerging modalities such as artificial intelligence-driven lesion detection. For now, the study by Rodrigo et al underscores the need for wider adoption and improved CE training to improve the quality of IBD surveillance.

**Publication note**

Letters to the editor do not necessarily represent the opinion of the editor or publisher. The editor and publisher reserve the right to not publish letters to the editor, or to publish them abbreviated or in extracts.

## References

[1] Motta RV Gupta V Hartery K Dye-based chromoendoscopy detects more neoplasia than white light endoscopy in patients with primary sclerosing cholangitis and IBD Endosc Int Open 2024 12 E1285 E1294 39534278 10.1055/a-2437-8102 PMC11555309

[2] Mohamed MFH Marino D Elfert K Dye chromoendoscopy outperforms high-definition white light endoscopy in dysplasia detection for patients with inflammatory bowel disease: An updated meta-analysis of randomized controlled trials Am J Gastroenterol 2024 119 719 726 38038351 10.14309/ajg.0000000000002595

[3] Forbes N Boyne DJ Mazurek MS Association between endoscopist annual procedure volume and colonoscopy quality: Systematic review and meta-analysis Clin Gastroenterol Hepatol 2020 182192-2208.e12 10.1016/j.cgh.2020.03.046 32240836

[4] Al Bakir I Kabir M Yalchin M Optimising inflammatory bowel disease surveillance and dysplasia management-Where do we stand? United European Gastroenterol J 2022 10 1054 1062 10.1002/ueg2.12330 PMC9752268 36349435

[5] Choi CR Al Bakir I Ding NJ Cumulative burden of inflammation predicts colorectal neoplasia risk in ulcerative colitis: a large single-centre study Gut 2019 68 414 422 10.1136/gutjnl-2017-314190 29150489 PMC6581019

